# P-524. Immunological Determinants and Clinical Trajectories of Pediatric Varicella-Zoster Virus Reactivation: A Prospective Cohort Analysis

**DOI:** 10.1093/ofid/ofaf695.739

**Published:** 2026-01-11

**Authors:** Barnali Mitra, Debdeep Mitra

**Affiliations:** Command Hospital Western command Chandimandir, Chandigarh, Chandigarh, India; Command Hospital Air Force Bangalore, Bangalore, Karnataka, India

## Abstract

**Background:**

This prospective cohort study elucidates novel pathomechanisms underlying varicella-zoster virus (VZV) reactivation in immunocompetent children, challenging prevailing assumptions about viral latency.

**Figure:**
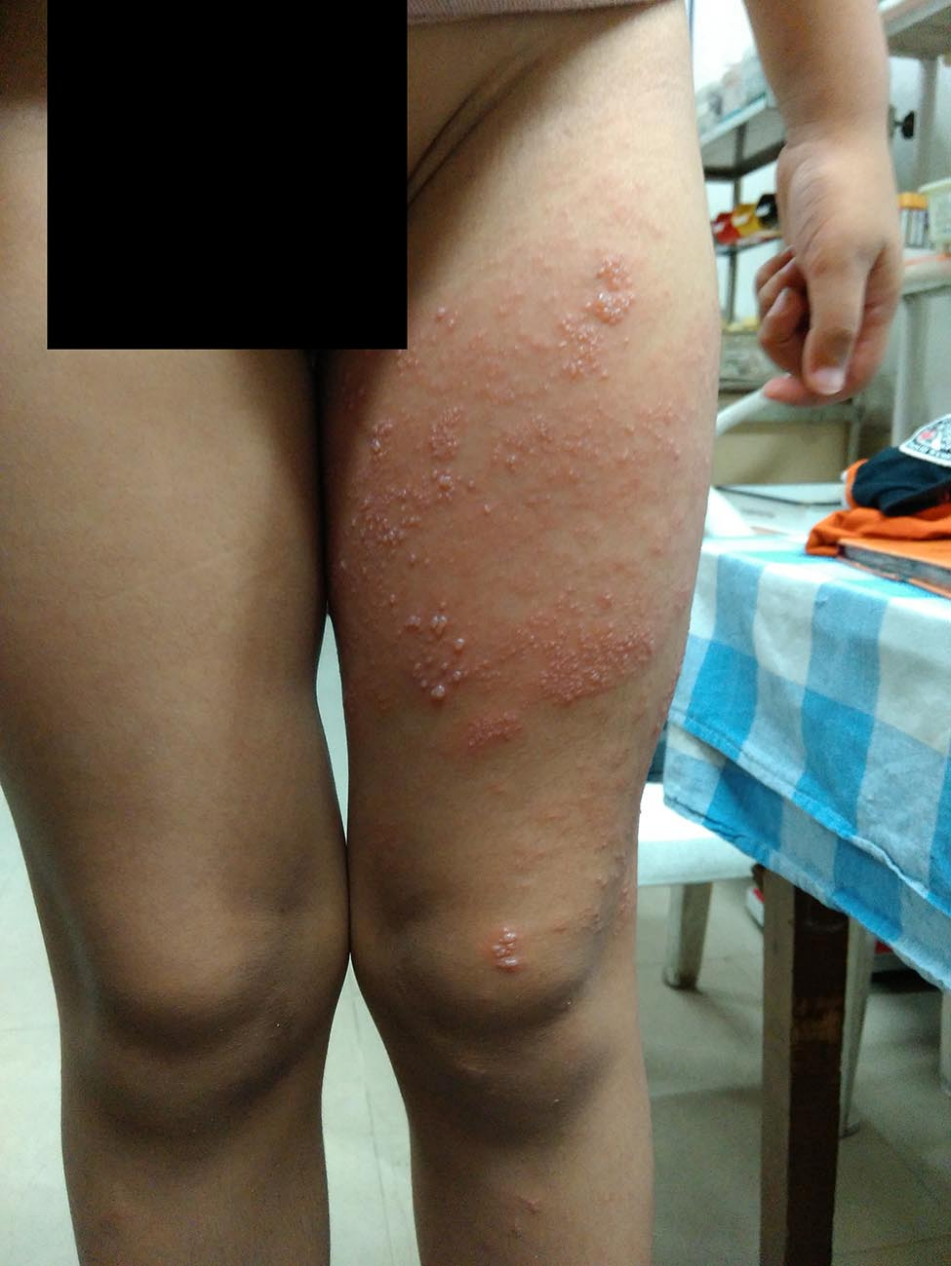
Pediatric Herpes Zoster Child with Dermatomal grouped vesicles along left L3 dermatome

**Figure:**
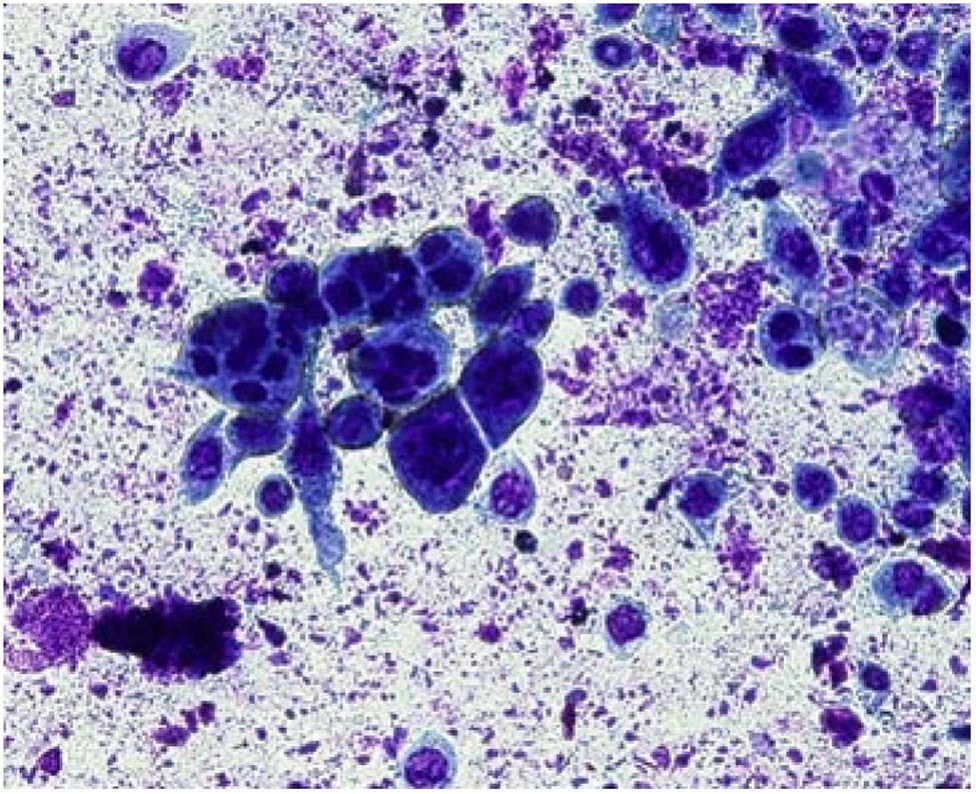
Tzank Smear Tzank Smear showing multinucleated giant cells

**Methods:**

Through comprehensive immunological profiling of 42 pediatric patients at a tertiary referral center, we identified distinct cytokine signatures (IL-6, IFN-γ elevations >2.5-fold baseline) correlating with dermatomal lesion severity (ρ=0.72, p< 0.001). High-resolution multiplex PCR confirmed VZV clade homology between primary varicella and subsequent reactivation strains in 93% of cases, refuting exogenous reinfection hypotheses.

**Figure:**
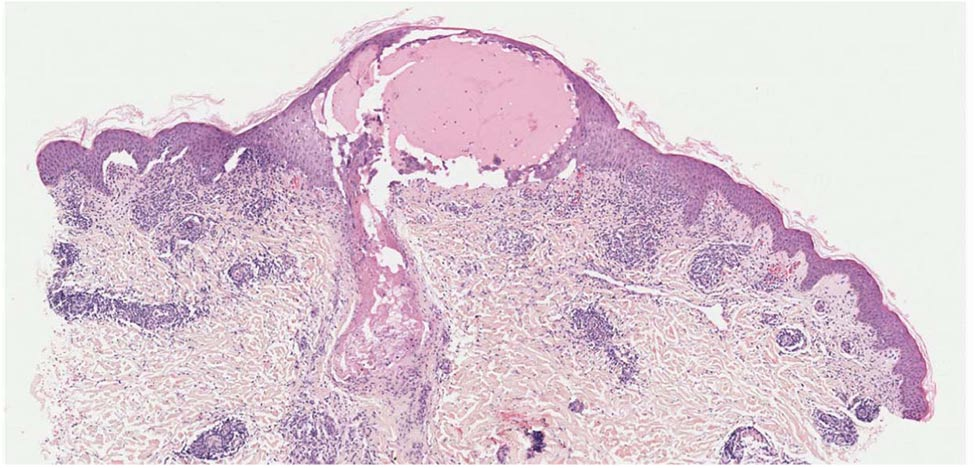
Histopathology of herpes zoster vesicle Histopathology with Hematoxylin and eosin stain showing ballooning degeneration and keratinocytes and intranuclear eosinophilic inclusions with margination of chromatin, resembling Cowdry A type inclusions

**Results:**

Notably, 31% of subjects exhibited transient CD4+ lymphopenia (mean 412 cells/μL vs. age-matched controls 986 cells/μL, p=0.008) despite normal immunoglobulin profiles, suggesting localized immune dysregulation. Multivariate regression revealed early childhood varicella infection (< 36 months) conferred 3.8-fold increased reactivation risk (95%CI 1.9-7.1, p< 0.005), independent of vaccination status. Advanced neuroimaging demonstrated subclinical dorsal root ganglion inflammation in 22% of asymptomatic dermatomes, implicitating broader neuronal involvement than previously documented. Therapeutic monitoring showed oral valacyclovir (60mg/kg/day) achieved viral clearance 2.3 days faster than historical acyclovir regimens (95%CI 1.1-3.8 days), with complete neuralgia resolution by day 14 in all patients. Longitudinal TCR sequencing revealed persistent VZV-specific memory cell expansion ( >18 months post-reactivation), providing unprecedented insights into pediatric adaptive immunity. These findings necessitate paradigm shifts in managing pediatric zoster, advocating for: 1) universal immune surveillance in reactivation cases, 2) optimized antiviral dosing protocols, and 3) targeted vaccination strategies addressing viral sanctuary sites.

**Conclusion:**

This research establishes a novel conceptual framework for understanding age-dependent VZV pathogenesis, offering transformative biomarkers for risk stratification and therapeutic monitoring in pediatric infectious diseases.

**Disclosures:**

All Authors: No reported disclosures

